# Moderating Role of Initiative on the Relationship Between Intrinsic Motivation, and Self-Efficacy on Entrepreneurial Intention

**DOI:** 10.3389/fpsyg.2022.866869

**Published:** 2022-10-12

**Authors:** Francisca N. Ogba, Kalu T. U. Ogba, Lawrence E. Ugwu, Nkechi Emma-Echiegu, Adaobi Eze, Solomon Amaechi Agu, Bernard Akonam Aneke

**Affiliations:** ^1^Department of Educational Foundations, Alex Ekwueme Federal University Ndufu Alike Ikwo, Abakaliki, Nigeria; ^2^Department of Psychology, University of Nigeria Nsukka, Nsukka, Nigeria; ^3^Department of Psychology, Renaissance University, Enugu, Nigeria; ^4^Department of Psychology, Coal City University Ugbawka, Enugu, Nigeria; ^5^Department of Psychology and Sociological Studies, Ebonyi State University, Ebonyi, Nigeria; ^6^Department of Psychology, Enugu State University of Science and Technology, Enugu, Nigeria

**Keywords:** initiative, intrinsic motivation, self-efficacy, entrepreneurial intention, graduate

## Abstract

The growing population of new graduates and the increasing scarcity of employment opportunities have made entrepreneurship an unavoidable option for employment and self-sustenance. This study investigates the effect of the initiative in moderating the relationship between intrinsic motivation, self-efficacy, and entrepreneurship intention through the integrated framework of theory of planned behaviour, self-determination, and humanism. This study contributes insights to how these factors moderated by initiative influence entrepreneurial intention among graduating students of tertiary institutions in Nigeria. This study adopted a cross-sectional design to examine the moderating role of initiative on the relationship between intrinsic motivation, and self-efficacy on entrepreneurial intention among Nigerian graduates. A total number of 688 graduate students, including 266 (38.6%) males and 422 (61.4%) females with a mean age of 24.30 years (SD = 3.69), participated in the study. Participants responded to a self-report questionnaire containing Initiative, Intrinsic motivation, self-efficacy, and entrepreneurial intention scales. Results showed that all the variables correlated positively with entrepreneurial intention. Furthermore, initiative moderated the relationship between self-efficacy and entrepreneurial intention [value and start-ups/operations (OPS)], such that high self-efficacy with high initiative showed higher entrepreneurial intention (value). While to those with low self-efficacy and low initiative, high self-efficacy with high initiative showed higher entrepreneurial intention (OPS) compared to low self-efficacy and low initiative. The study highlighted the role of initiative in transforming young graduates’ entrepreneurial intention into full-fledged entrepreneurs.

## Introduction

There has been growing interest in promoting and supporting entrepreneurship as an attractive alternative to waged employment among students around the globe (Gelard and Saleh, 2010; Ahmed et al., 2020). Like most developing nations, Nigeria is experiencing significant economic challenges that include unemployment of her teeming youths, which presents the need for entrepreneurial skills to be acquired and used to curb this menace (Obschonka et al., 2017). As a result, considerable attention has been devoted to understanding the determinants of an individual’s decision to engage in entrepreneurial activities (Dvouletý and Orel, 2019). As a result, today, entrepreneurship has become one of the most vital forces in developing nations and reinforces the world’s economic growth.

Entrepreneurial activities are perceived as instruments that propel nations’ long-term economic growth (Urbano et al., 2020). In support, the recent research literature has focused on the role of intention within the entrepreneurial process (van Gelderen et al., 2018; Abbasianchavari and Moritz, 2019; Hou et al., 2019). Hence intents and the formation process are now considered within the entrepreneurship literature. The link between intention and behaviour is very well explained in psychology. For example, it is believed that intention mirrors the factors that motivate behaviour, and it is a reliable indicator of how hard a person is willing to try and how much effort they make to achieve a behaviour (Ajzen, 1991). As a result, the intention is widely seen as a powerful predictor of behaviour, especially in purposive, planned, and goal-oriented behaviour (ElHaffar et al., 2020). Entrepreneurial behaviour is typically seen as an intentional behaviour directed towards specific entrepreneurial events, such as creating a new business, company, or product. These intentions are seen as central in understanding the entrepreneurial intents developed from rational and intuitive thinking, which are affected by the entrepreneur’s social, political, and economic context and their perceived history, current personality, and abilities (Zaremohzzabieh et al., 2019). Entrepreneurial intention is defined as the intention toward starting a high-growth business, the industry, or other service rending ventures (Ozaralli and Rivenburgh, 2016; Nikou et al., 2019).

The present economic challenges in Nigeria, where we have a high unemployment rate of 33.3% (National Bureau of Statistics, 2021), and are now heightened by the scourge of the coronavirus pandemic, have served as an eye-opener to students and graduates. Students need to begin to look for opportunities that will create economic and financial gain for them in the future. Questions on those intrinsic factors that may serve as a functional urge for personal success and exploitation of valuable business opportunities in this global world of the quest for employment among Nigerian graduates are central in this research. This study takes a stand on the relationship between intrinsic motivation and entrepreneurial intention towards the chance for personal success. Intrinsic motivation can be traced by a causal agent of action known as the drive. The drive is a complex factor associated with biological, instinct, and tension reduction (Joshua, 2014). Often, in human motivation, individuals tend to be aroused by external stimuli. This study tried to explore the effect of initiative in the relationship between intrinsic motivation, self-efficacy, and entrepreneurial intention among young Nigerian graduates.

## Intrinsic Motivation and Entrepreneurial Intention

Behaviour is intrinsically motivated when it is freely done due to an aroused interest in a specific task, with the satisfaction expected from it. Michael and Doris (2001) assert that the hallmark of intrinsic motivation is the experience of positive affect associated with it, which includes: enjoyment, happiness, satisfaction, and pleasure. Self-determination theory also explained that three primary needs drive intrinsically motivated individuals; the need for self-autonomy; the need to exercise competence; and the need to connect with others (Joshua, 2014). Deci and Ryan (2000) supported this and added that a high perception of achievement motives promotes the degree of self-autonomy (i.e., the self as an agent of action).

Invariably, humanistic theory explained intrinsic motivation to be associated with an external schema. Maslow (1968, 1970) asserts that motives for behaviour are characterized by a physiological to psychological continuum. Humans tend to satisfy the most intrinsic need of survival, then critical needs for an idealized self. Locke (1968) also added that motives for behaviour orientation towards achieving cognitive and affective homeostasis.

Research has also shown that intrinsically motivated goal pursuits individuals are related to potentiating perceptions, internalized behaviours, and activities that obliged a balance between challenge and optimism (Li et al., 2020) Thus, if an intrinsically motivated goal pursuit individual can be found to have optimistic potentiality and internalized behaviours towards activities that engender a balance between challenge and ability, it is alleged that intrinsic motivation could be related to entrepreneurial intention.

According to Escolar-Llamazares et al. (2019), entrepreneurship is related to innovation, productivity, growth, creation of employment, and personal success. However, the entrepreneurial intention is much more of the individual, and it is one of the most integral parts of the entrepreneurship process (Bulsara et al., 2010). To be an entrepreneur is an activity that requires an individual’s reasoned action because of its complex behavioural discovery of opportunity and exploitation. Ajzen (1991) theory of planned behaviour explained that the focal construct of the theory of planned behaviour (TPB) is the individual’s tendency to exploit an opportunity. Studies (Hassi, 2016; Garrido-Yserte et al., 2019; Cardella et al., 2020; Ndofirepi, 2020), have been conducted on the quality of exposure and predisposition of an entrepreneurial tendency to students through entrepreneurship education. In various learning institutions, and how it predicted students’ mindset and intention to start accomplishing a business.

For instance, Ratten and Usmanij (2020) asserts that entrepreneurship education has paved the way toward giving people the tendency to perceive business as an opportunity for economic and financial gain, and the general information required is provided to them. This, therefore, propels their intention and dispositions to establish a business venture. Agboola (2020) also found that entrepreneurship education has propelled personal factors during the training programme that enable students to change their perspective from looking for a job to creating job opportunities by building up their potentials and abilities. Ogundele et al. (2012) found out that education changes people’s mindset, self-efficacy, and attitude to become an entrepreneur. Zollo et al. (2021) supported those intuitions play many roles in building entrepreneurial intention for their students. Even the Federal government of Nigeria has it as a mandate that every institution of learning should instil entrepreneurship culture to their student, and this direction was given to the Nigerian National University Commission (NUC) in 2014.

*H1a: Intrinsic Motivation will significantly predict Entrepreneurial Intention (value)*

*H1b: Intrinsic Motivation will significantly predict Entrepreneurial Intention (start-ups/operations)*

## Self-Efficacy and Entrepreneurial Intention

Self-efficacy, which is the belief that people can produce desired effects that are central to most human functioning, might be a predictor of entrepreneurial pursuits (Santos and Liguori, 2020) Self-efficacy has been found to have direct effects on business growth (Baum and Locke, 2004). Shaker (2018) argued that for individuals to develop a desire to venture into entrepreneurship, they need to possess abilities like risk-taking, innovativeness, achievement-oriented, and can-do mentality (self-efficacy). The self-doubt of an individual about their abilities can withhold the individual from engaging in an entrepreneurial journey. More so, the belief in oneself is more important than even possessing them and any experience of the task (Shaker, 2018).

Entrepreneur’s self-efficacy is defined as a person’s belief in their ability to launch an entrepreneurial venture successfully (Liu et al., 2019) Entrepreneurial self-efficacy is viewed as having the capabilities that can modify a person’s belief in their likelihood of completing the tasks required to successfully initiate and establish a new business (Bandura, 1986). According to Saraih et al. (2018), self-efficacy positively influences entrepreneurial intention. It can be enhanced by training and education to improve the decision-making processes of the entrepreneurs (Liu et al., 2019) and is the best variable to predict an entrepreneur’s performance (McGee and Peterson, 2017). In their study, Ogba et al. (2019) examined the role of personality and self-efficacy in entrepreneurial intention as a capacity for venturing into business. Their findings showed that openness to experience and self-efficacy were factors in entrepreneurial intention among undergraduate students. Several other studies (e.g., Rosique-Blasco et al., 2017; Mei et al., 2017) have focused more on entrepreneurial self-efficacy than self-efficacy itself. Therefore, we assumed that entrepreneurial self-efficacy is conceptually more closely related to entrepreneurial intention than general self-efficacy. We, therefore, wanted to know whether self-efficacy (as a factor), which had received less empirical attention, could also be related to entrepreneurial intention. Those were the gaps in the literature that our study intended to fill.

*H2a: Self-efficacy will significantly predict Entrepreneurial Intention (value)*

*H2b: Self-efficacy will significantly predict Entrepreneurial Intention (start-ups/operations)*

## Initiative and Entrepreneurial Intention

Personal initiative is another variable considered in this research as a moderator of the relationship between intrinsic motivation, self-efficacy, and entrepreneurial intention. How a person may perceive the worth of being an entrepreneur could affect their choice to be an entrepreneur. According to Cheng et al. (2020) Initiative is a cognitive schema comprised of different human actions triggered by external forces or motives. It is a set of connected urges and capabilities that formed an intent and desire to create a job, overcome difficulties, and persist in it (Eugene, 2015). Using Schwartz’s theory of value, initiative is assumed to be a need-directed action that serves as a guiding principle for individuals (Schwartz, 1992; Schwartz et al., 2012).

**FIGURE 1:**
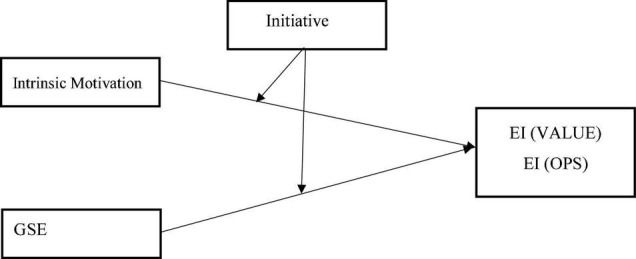
Conceptual model.

It is a self-starting nature and persistent in overcoming difficulties that may arise in pursuing a goal, according to Frese et al. (1997). It is suspected to moderate individuals’ intrinsic motivation and self-efficacy with entrepreneurial intention, given that initiative involves developing ideas and taking charge of an idea, whether big or small. Initiative has been conceptualized as an active and proactive approach to changes in the presence of difficulties, developing of plans for dealing with future challenges, and responding to environmental demands (Frese and Fay, 2001). It, however, involves a self-starting goal; persistent; and proactivity and may moderate the degree of intrinsic motivation, and self-efficacy with entrepreneurial intention due to the following suspected reasons: first, a self-starting goal, some students or graduates may have the will to start a venture which may seem challenging to do by others, yet ignoring all challenges pushed their way further to take unusual actions/steps to establish a recruiting business. Second, by being persistent, students or graduates may try to continue their intention to be entrepreneurs and protect the ideal information from barriers and threats. Finally, in being proactive, the student or graduate may be alert to monitor any unlikely problems and difficulties that may interrupt their entrepreneurial intention. However, the moderating role of initiative on the relationship between intrinsic motivation, and self-efficacy on entrepreneurial intention is further illustrated in a model as shown in Figure 1.

The conceptual model explains that initiative moderates the relationship between intrinsic motivation, self-efficacy, and the two dimensions of entrepreneurship intention. Initiative, which is the actual action, or the step taken to engage in a behaviour, helps explain intrinsic motivation (Intrinsic motivation involves engaging in an activity for the pleasure and satisfaction derived from it). On the other hand, self-efficacy-how well, one can execute a course of action required to deal with prospective situations. The general idea is that initiative is expected to increase intrinsic motivation and self-efficacy in predicting entrepreneurial intention. In other words, we proposed that graduate students who have the initiatives may likely transform their entrepreneurial intention to become full-fledged entrepreneurs only if they are internally motivated. Thus, the study sought to find out as follows:

*H3a: Will initiative significantly predict Entrepreneurial Intention (value)*

*H3b: Will initiative significantly predict Entrepreneurial Intention (start-ups/operations)*

*H4a: Will initiative moderate the relationship between intrinsic motivation and Entrepreneurial Intention (value)*

*H4b: Will initiative moderate the relationship between intrinsic motivation and Entrepreneurial Intention (start-ups/operations)*

*H5a: Will initiative moderate the relationship between self-efficacy and Entrepreneurial Intention (value)*

*H5b: Will initiative moderate the relationship between self-efficacy and Entrepreneurial Intention (start-ups/operations)*

## The Present Study

No doubt that the domain of entrepreneurship had witnessed an influx of research endeavours. Some of which studied Universities supporting students’ entrepreneurship tendency (Adelekan and Tijani, 2017; Ahmed et al., 2020; Leung et al., 2020), entrepreneurial alertness for opportunity identification (Kevin, 2016), career adaptability in predicting entrepreneurial intention including those with special needs (Laramie et al., 2014; Leung et al., 2020). Related work in this domain was done by Michael and Doris (2001), who examined personal initiative (PI) as an active performance concept for work in the 21st century. According to them, PI was measured within the framework of a situational interview (Latham and Saari, 1984), unlike our present study that examined initiative from a moderating lens. As against our study, a more recent study by (Escolar-Llamazares et al., 2019) examined entrepreneurial interest among Spanish youth. They studied the direct antecedent of entrepreneurial intention, and its relation with various socio-educational, psychological, and health-related variables that influence choice. In the same vein, Ogba et al. (2019) examined the role of personality and self-efficacy in entrepreneurial intention as a capacity for venturing into business. Though very similar to our present study, it did not consider the moderation effect of initiative. Our study becomes germane because of the global search for employment amidst a high rate of unemployment.

Our study is also expected to be an eye-opener to young graduate students towards developing initiative and creativity as their academic training will help actualize their entrepreneurial intention. Moreover, our study is apt given that the Nigerian educational sector releases thousands of student graduates yearly into the labour market without provisions for their employment. Therefore, we reasoned that in the high rate of Nigerian unemployment, these young graduates should be taught that initiative is required to catapult their entrepreneurial intention into becoming self-employed. Hence, it seems young student graduates are yet to tap this idea which has also not been empirically documented (to the researchers’ knowledge).

Entrepreneurship has been variously studied. However, there are much more research gaps that need to be filled. Such research gap as considering how entrepreneurship intention could be facilitated by the act of taking concrete initiative to actualize the desired goal. Initiative is studied as a moderator in the relationship between intrinsic motivation, and self-efficacy on entrepreneurial intention is very epic. Against this backdrop, we hypothesized that intrinsic motivation and self-efficacy would significantly predict entrepreneurial choice among graduate students; Initiative will substantially moderate the relationship between intrinsic motivation and entrepreneurial intention among graduate students.

## Materials and Methods

### Participants and Procedure

A total number of 688 graduates who were under the compulsory National Youth Service Corps (NYSC) in two Nigerian States (Ebonyi and Enugu) participated in the study. NYSC is a compulsory exercise for all fresh graduates below 30 years, randomly posted to other states in Nigeria other than their States of origin and tertiary education, except on medical or marital grounds. The scheme was initiated in 1974 to encourage national integration. The majority of the participants were usually posted to teach in schools regardless of their academic endeavour. For this study, the participants were selected at their respective Orientation Camps where they were accommodated for three weeks’ orientation at a secluded location. Those who volunteered after reading and giving their consent were administered the scale. Their ages ranged from 21 to 29 years (M = 24.30; SD = 3.69). They consisted of 266 (38.6%) males and 422 (61.4%) females. Six hundred and ten (88.66%) were single while 78 (11.34%) were married. The scale took an average participant 15 min to complete.

### Instruments

#### Intrinsic Motivation

Intrinsic motivation and extrinsic motivation were measured using Oliver and Anderson’s (1994). Examples of items are “I do not need a reason to sell; I sell because I want to” and “When I perform well, I know it is because of my desire to achieve.” The scale was adapted for this study with a reliability of. 82. Response scales are 1–7 (1 = Strongly Disagree – 7 = Strongly Agree). See Table 1 for reliability and validity.

**TABLE 1:** Internal consistency reliability and convergent validity.

Construct	Measurement items	Loading	C R	C A	AVE
Intrinsic motivation	s1	0.594	0.588	0.597	0.511
	s2	0.459			
	s3	0.482			
	s4	0.442			
	s5	0.462			
	s6	0.599			
GSE	l1	0.608	0.728	0.718	0.606
	l2	0.630			
	l3	0.568			
	l4	0.560			
	l5	0.555			
	l6	0.409			
Initiative	f1	0.567			
	f2	0.450	0.665	0.689	0.546
	f3	0.552			
	f4	0.415			
	f5	0.592			
	f6	0.495			
	f7	0.670			
EI (value)	sl1	0.626	0.597	0.648	0.618
	sl2	0.627			
	sl3	0.738			
	sl4	0.772			
	sl2	0.536			
EI (OPS)	sl11	0.492	0.677	0.638	0.663
	sl12	0.696			
	sl13	0.661			
	sl14	0.646			

*Note: GSE = Generalised self-efficacy; EI = Entrepreneurial Intention; CR = Composite Reliability; CA = Cronbach’s Alpha; AVE = Average Variance Extracted.*

#### Self-Efficacy

Self-efficacy was measured with a 21-item self-efficacy scale. The scale was developed by Schwarzer and Jerusalem (1993) to assess the general sense of perceived self-efficacy to predict coping with daily hazards and adaptation after experiencing all kinds of stressful life events. The scale has a Likert response format ranging from 1- “Not at all true” to 4- “Exactly true.” A sample item is “I feel responsible for my own life”—Table 1 for reliability and validity.

#### Initiative

Initiative was measured using Beckmann and Kazén’s (1994) initiative. The scale contains seven items that describe a particular situation. Each item has two alternative answers (A or B). One answer corresponds to initiative and the other to hesitation. In the following example, “A” corresponds to hesitation, and “B” corresponds to initiative: “When I have to take care of something important that is also unpleasant: (A) I do it and get it over with, or (B) It can take me a while before I can bring myself to it.” A score of one is assigned to responses that correspond to initiative and zero to hesitation. Scores are added to create a continuous variable, with larger scores denoting a higher initiative, initiative ranges from zero to eight. The researchers conducted a reliability and validity test (see Table 1).

#### Entrepreneurial Intention

Entrepreneurial intention was measured by an 8-item entrepreneurial intention scale developed by Valliere (2015). The scale has two sub-scales: value and start-ups/operations (ops); value is the creation of value in the market, e.g., of item “Develop a prototype of a product/service” and “Test my value proposition in the market.” While ops are the practical start-up and operations of an entrepreneurial venture, e.g., “Invest my own resources into my business” and “Open a business bank account.” The author reported a reliability index of.89 for value and.82 for ops. See Table 1 for reliability and validity.

#### Statistics/Design

The study was a cross-sectional design. Descriptive statistics (mean and SDs) and partial correlation coefficients between variables were examined. Smart PLS 3.1 was used to assess the reliabilities, validity of the instruments. The Structural Equation modelling and the moderation were tested.

#### Measurement Models

The measurement model was tested to assess the internal consistency reliability, convergent validity (CV), and discriminant validity (DV) of the constructs used in this study. Internal consistency reliability measures the degree to which the items measure the latent constructs (Hair et al., 2014a; Ramayah et al., 2016). Composite reliability was assessed as a measure of internal consistency (Hair et al., 2017). The measurement model with composite reliability above the threshold value of 0.7 for each construct is considered satisfactory (Nunnally, 1978; Nunnally and Bernstein, 1994; Richter et al., 2016). DV is the degree to which a construct is distinct from other constructs in the model (Hair et al., 2017). We used two methods to assess DV; first, we used the Fornell and Larcker (1981) criterion – comparing the correlation between the constructs and the square root of the Average Variance Extracted (AVE) for that construct. To achieve DV, the square root of the AVE for each latent variable must exceed the correlation value for the same construct (Fornell and Larcker, 1981). As shown in Table 1, the results indicate adequate DV, with the AVE square root values higher than the correlation values in the rows and columns (Fornell and Larcker, 1981).

## Results

Before analysing the structural model, in addition to reliability and validity, the variance inflation factor (VIF) was assessed to compute for multicollinearity. A VIF value greater than 10.0 is regarded as an indication of multicollinearity (Burns and Burns, 2008). However, Hair et al. (2014b) recommends a cut-off value of 5.0 for multicollinearity. The VIF results for each construct, which were1.033–1.663, are all below the threshold value of 5.0, indicate that collinearity issues between the constructs were absent from this study (see Table 1).

Table 2 reports the means, standard deviations, and correlations among the variables. Among the demographic variables (i.e., age and gender) only age was significantly correlated with initiative (r = 0.098, p = 0.048). Self-efficacy (r = 0.323, *p* = 0.001), entrepreneurial intention (value) (r = 0.351, *p* = 0.001) and entrepreneurial intention (ops) (r = 0.361, *p* = 0.001). While self-efficacy had a significant correlation with entrepreneurial intention (value) (r = 0.378, *p* = 0.001) and entrepreneurial intention (ops) (r = 0.435, *p* = 0.001). But initiative had no significant relationship with all other variables except age (r = 0.098, *p* = 0.048), entrepreneurial intent (value) (r = 0.363, *p* = 0.001) and entrepreneurial intent (ops) (r = 0.338, *p* = 0.001).

**TABLE 2:** Descriptive statistics and correlation matrix.

		M	SD	1	2	3	4	5	6	7
1	Age	24.290	3.685	1	0.073	–0.025	–0.024	0.098*	–0.025	–0.027
2	Gender				1	–0.055	0.078	–0.064	0.027	–0.036
3	Intrinsic motivation	21.535	4.645			1	0.323**	–0.007	0.351**	0.361**
4	GSE	20.594	4.975				1	0.083	0.378**	0.435**
5	Initiative	3.678	1.485					1	0.363**	0.338**
6	EI (value)	14.342	3.627						1	0.520**
7	EI (ops)	15.144	3.584							1

*P <. 05; **p < 0.001; GSE = Generalized self-efficacy; EI = Entrepreneurial Intention.

From Table 2 above, all the variables related positively to the two dimensions of entrepreneurial intention. This indicates that as each variable increases, both dimensions increase.

### Structural Model Analysis

The second step in analysing using the PLS method is by assessing the structural model. The structural model is achieved by running the bootstrap resampling technique (Henseler et al., 2009) with 5000 iterations to ensure stability (Chin, 1998; Hair et al., 2014b). This provides analyses on hypotheses and constructs’ relationships based on examination of standardized paths. The result of our assessment is displayed in Table 3.

**TABLE 3:** Moderation coefficients, *t*-values, and support for the hypotheses.

	Value				OPS				Support for hypothesis
	PC	*t*-value	LLCI	ULCI	PC	*t*-value	LLCI	ULCI	
H4: Intrinsic motivation - > Initiative	0.051	0.044^ns^	–0.042	0.051	–0.265	1.832^ns^	–0.004	0.087	Rejected
H5: GSE- > Initiative	0.236	2.227**	0.055	0.145	–0.143	2.193*	0.011	0.099	Supported

*p < 0.05; **p < 0.001; PC = Path Coefficient; n.s. = not significant.

Table 4 showed the moderation result, indicating that initiative moderated the relationship between GSE and entrepreneurial intention (value and ops).

**TABLE 4:** Hypotheses testing results.

Relationship	R^2^	Estimate	*t*-value	Hypothesis supported
EI (value)	0.441			
H1a: Intrinsic motivation = > (+)Value		0.356	10.8**	Supported
H2a: GSE = > (+)Value		0.206	5.083**	Supported
H3a: Initiative = > (+)Value		0.113	5.315**	Supported
EI (OPS)	0.522			
H1b: Intrinsic motivation = > (+) OPS		0.296	10.728**	Supported
H2b: GSE = > (+) OPS		0.334	9.316**	Supported
H3b: Initiative = > OPS		0.141	5.020**	Supported

**p < 0.001; GSE = Generalized self-efficacy; EI = Entrepreneurial Intention.

## Discussion

Entrepreneurial activities perceived as instruments that propel nations’ long-term economic growth (Romer, 1994); have become one of the most vital forces in developing countries and reinforce the world’s economic growth. The current study advanced literature on the entrepreneurial intention by examining initiative as a moderator underlying the relationship between intrinsic motivation, self-efficacy, and entrepreneurial intention (value and ops). Empirical evidence indicated that economic challenges like the teeming youths’ unemployment require entrepreneurial skills to curb the menace (Nwankwo, 2011). The need for entrepreneurship is precisely the case in Nigeria at this point. With its nascent finding, the present study provided evidence to the above postulation confirming hypotheses 1a and 1b. Furthermore, it indicated that intrinsic motivation is positively related to entrepreneurial intention (value and ops). This finding suggests that new graduates with the feeling of being intrinsically motivated have more likelihood of perceiving that their entrepreneurial intention is important to their society.

Similarly, graduates with higher intrinsic motivation experience, are more likely to engage practically in implementing their idea. Therefore, there is no doubt that intrinsic motivation is a strong force that propels individuals into entrepreneurial intention and engagement. In agreement with our findings, earlier studies such as Agboola (2020) showed that entrepreneurship education has a propelling personal factor that enables students to change their perspective from looking for a job to creating job opportunities by building up their potential and abilities. This implies that entrepreneurship offers students the opportunity to look inward and discover many good capabilities and device ways of utilizing them. Ogundele et al. (2012) gave more support to the present finding, whose finding indicated that education changes people’s mindsets and attitudes to become entrepreneurs. Zollo et al. (2021) also supported those intuitions play many roles in building entrepreneurial intention in students. Entrepreneurship involves intuitive thought processes that enable individuals to discover new and solve problems within society.

According to Joshua (2014), in self-determination theory, intrinsically motivated individuals are driven by three primary needs: self-autonomy, the need to exercise competence, and the need to connect with others. All these qualify as characteristics of an entrepreneur. In agreement, Deci and Ryan (2000) supported this. They added that a high perception of achievement motives promotes the degree of self-autonomy (i.e., the self as an agent of action).

Our study equally made nascent discoveries on the aspect of self-efficacy and entrepreneurial intention. The results also showed that self-efficacy was positively and significantly related to the two dimensions of entrepreneurial intention (value and ops). Hypotheses 3a and 3b are therefore confirmed. This suggests that students or new graduates who have higher self-efficacy are likely to perceive that their entrepreneurial intention has value to their society. One good reason for this is that students and recent graduates believe in their ability to evaluate their environment and what they need to add value to it. There is the idea that since students and new graduates have found an essential need in their environment and believe that they can produce desired effects (Bandura, 2000), they have the confidence to undertake the practical task of establishing the needed venture to address the need. Chen et al. (1998) buttressed Bandura’s (2000) postulation and concluded that belief in oneself is more important than even possessing them and any task experience. Hence, a person’s confidence in their ability to successfully launch an entrepreneurial venture (McGee et al., 2009) is all about the entrepreneur’s self-efficacy. Extant findings by Saraih et al. (2018) showed that self-efficacy positively influences entrepreneurial intention. In the words of McGee et al. (2009), it can be enhanced by training and education to improve the decision-making processes of the entrepreneur, and is one best variables to predict an entrepreneur’s performance (Shane et al., 2003).

Another interesting finding of our present study is that initiative is positively related to entrepreneurial intention (value and ops). Students/new graduates who perceived themselves as self-starters tend to see that their entrepreneurial intention is of more excellent value to their society and equally take charge of their ideas to start up the business regardless of the difficulty perceived or encountered (Schwartz et al., 2012; Eugene, 2015). Entrepreneurship involves the development of the ability to initiate and introduce novel ideas. Therefore, when students/new graduates are trained with relevant knowledge on generating and introducing new ideas on business and job creation, they become more entrepreneurial. In support of this, Eugene earlier maintained that initiative is a set of connected urges and capabilities that formed an intent and desire to create a job, overcome difficulties, and persist in it (Eugene, 2015). Cheng et al. (2020) added that it is a cognitive schema comprising different human actions triggered by external forces or motives. According to Schwartz’s theory of value, the initiative is a need-directed action that serves as a guiding principle for individuals (Schwartz, 1992; Schwartz et al., 2012).

Although initiative as shown earlier, is significantly related to entrepreneurial intention (value and ops), contrary findings of the study showed that it did not moderate the relationship between intrinsic motivation and entrepreneurial intention (value and ops). We postulated that since the initiative is a self-starting nature and persistent, it is overcoming difficulties in pursuing a goal (Frese et al., 1997). Our finding could be a result of the lack of relationship between intrinsic motivation and initiative. They could be described as independent of each other or that the relationship between them is not linear. The finding undoubtedly contradicts the mere perception that intrinsically motivated individuals ought to be high in initiating business ideas and ways of maintaining or improving on them.

**FIGURE 2:**
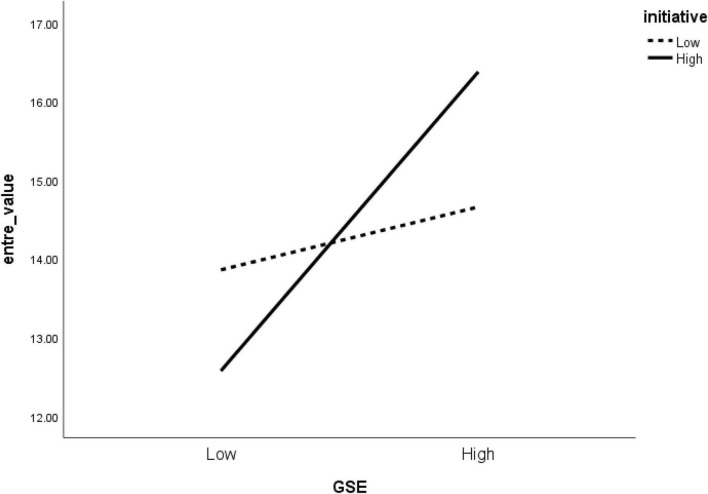
Moderating effect of initiative on the relationship between GSE and entrepreneurial intention (value).

**FIGURE 3:**
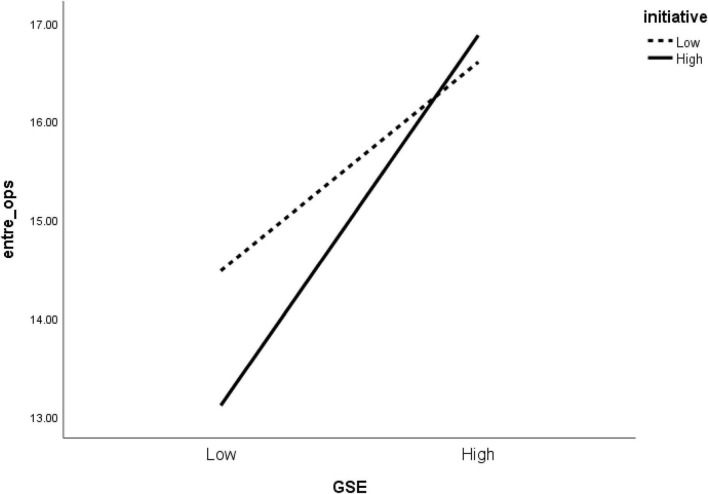
Moderating effect of initiative on the relationship between GSE and entrepreneurial intention (ops).

Initiative moderated the effect of self-efficacy on the two dimensions of entrepreneurial intention significantly. As shown in Figure 2, individuals with high GSE with high initiative showed higher entrepreneurial intention (value) compared to low GSE and low initiative individuals. Similarly, and as indicated in Figure 3, individuals of high GSE with high initiative showed higher entrepreneurial intention (ops) than low GSE and low initiative individuals. Earlier in the study, we followed the idea of researchers Frese et al. (1997) on initiative being a self-starting nature and persistent in overcoming difficulties that may arise in the pursuit of a goal and maintained that it is likely to moderate individuals’ self-efficacy relationship with entrepreneurial intention (value and ops). One good reason for this stems from the fact that initiative involves developing ideas and taking charge of an idea, whether big or small, and as conceptualized as an active and proactive approach to changes in the presence of difficulties, development of plans in dealing with future challenges, and responding to environmental demands (Frese and Fay, 2001). Another reason is that, in self-starting goals, some students or graduates may intend to start a venture that may seem challenging to others. Yet, they will ignore all challenges and push their way further to take unusual actions/steps to establish a recruiting business.

## Practical Implication

They have implications for humanity and society. First, entrepreneurship thrives in a society where individuals, especially young ones, grow in an environment that intrinsically develops and motivates them. Intrinsic motivation generates joy capable of making individuals think creatively to develop ideas that solve problems in society, as all entrepreneurial outputs are targeted at solving some societal issues. Intrinsic motivation can be better fueled, according to Ahmed et al. (2020). They proposed an entrepreneurship education structure where students are guided to engage in a practical task, conduct market analysis, pitch ideas, and present business plans. Entrepreneurial discoveries lie with having initiatives; the desired objective might be a mere academic exercise without active involvement. The Initiative introduces uniqueness and creativity in the thinking of entrepreneurs. Entrepreneurial education consequently influences students’ self-efficacy, making them believe in themselves and contribute to providing unique and lasting solutions to challenges faced in our societies. Individuals with the ability to generate novel ideas are more likely to trust themselves to develop businesses or ways of meeting societal needs and have more entrepreneurial intentions. Therefore, when government at all levels, educational or training institutions introduce programs, platforms, or activities to enhance students’ abilities to develop novel ideas and believe in themselves. Students will appreciate entrepreneurship and think more of ways of making society better than seeking jobs. A more significant percentage of students will become more job creators than seekers, and unemployment problems and challenges will be hugely solved.

The practical entrepreneurial initiative could significantly improve students’ experiential learning (Yeo and Marquardt, 2015; Corriveau, 2020). From experience, students learn to take up leadership roles and feel responsible for the outcome of their actions and inactions. The Initiative helps students make better decisions on engaging in entrepreneurial ventures.

### Limitations of the Study and Suggestions for Further Research

This work is, however, not without its limitations. Our theoretical framework draws from four rather distinct streams of research: entrepreneurial intention, initiative, self-efficacy, and motivations. Empirically, this work seeks to explore associations and does not claim any causality: the cross-sectional nature of the data does not, at this stage, allow further investigation of the presence of a direction of the relationship. The study points to the need for further investigations on why young graduates are mostly driven by intrinsic motivations in the entrepreneurial intention to create a spin-off but subsequently undertake the entrepreneurial action only when also pushed by extrinsic rewards. Young graduates are a particular set of potential entrepreneurs. Their habitual environment is somewhat different from the average potential entrepreneur’s, especially as they are entering a complex world of uncertainties because they are just leaving school. This issue requires further investigation, and this work represents a first step in highlighting the gap in the comprehension of the link between young graduate entrepreneurial intention, intrinsic motivation, and self-efficacy.

The hypotheses were tested with a quantitative study designed as an *ex ante*/*ex post* measurement. We suggest that further research study variables such as business planning activities, role models, student-oriented teaching, and feedback processes to ascertain their impact on increasing entrepreneurial intention.

## Data Availability Statement

The raw data supporting the conclusions of this article will be made available by the authors, without undue reservation.

## Ethics Statement

The studies involving human participants were reviewed and approved by Coal City University Enugu, Publication Ethics Committee. The patients/participants provided their written informed consent to participate in this study.

## Author Contributions

FNO contributed to the initial draft, review, and contributions. NE-E contributed to the data collection and analysis. BAA contributed to data collection. All authors contributed to the article and approved the submitted version.

## Conflict of Interest

The authors declare that the research was conducted in the absence of any commercial or financial relationships that could be construed as a potential conflict of interest.

## Publisher’s Note

All claims expressed in this article are solely those of the authors and do not necessarily represent those of their affiliated organizations, or those of the publisher, the editors and the reviewers. Any product that may be evaluated in this article, or claim that may be made by its manufacturer, is not guaranteed or endorsed by the publisher.
